# Excerpts

**Published:** 1890-07

**Authors:** John A. Miller

**Affiliations:** Professor of Medical Chemistry and Toxicology in the Medical Department of Niagara University


### Selections.

### EXCERPTS.

By JOHN A. MILLER, M. Sc., Ph. D.,

Professor of Medical Chemistry and Toxicology in the Medical Department of Niagara University.

BRIEGER'S TYPHOTOXINE. By L. De Blasi. (*Gazetta*, xviii., 521; *Jour. Chem. Soc.*, 1890, p. 321.) The author undertook a series of investigations to determine whether the ptomaine obtained by Brieger from the broth cultures of Eberth's bacillus is due to the specific action of that organism or only to the action of heat and acids on the albuminoids.

Pure cultures of *Eberth's bacillus* and of *micrococcus caudidans* were allowed to remain at a temperature of 37° for forty days, and then examined for alkaloidal substances.

The conclusions which the author arrives at are: That the method of direct extraction is valueless for the isolation of any definite alkaloid, but that Brieger's method is efficient, and that the negative result obtained from the culture of the non-pathogenic organism demonstrates that typhotoxine is either the direct product of the activity of Eberth's bacillus, or is due to the secondary action of heat and acids on some unstable substance produced by the bacillus. The results of inoculation with the substances extracted from the culture of *micrococcus caudidans* prove that the action of heat and acids was ineffectual to produce any toxic substance from it.

INFLUENCE OF INORGANIC SALTS ON DEVELOPMENT. By S. Ringer. (*J. Physiol.*, 11, 79; *Jour. Chem. Soc.*, 1890, 393.) The author has previously shown (*J. Physiol.*, 4, 5, 6, 7,) the necessity of inorganic salts for sustaining life and vital activity, in muscular contraction, the heart beat, the action of cilia, and the life of fishes. Of these calcium salts seem the most important, and there is antagonism between the action of these salts and those of sodium and potassium. An excellent fluid for sustaining the heart's beat is made by mixing suitable small proportions of the salts of these metals; and of the calcium salts the phosphate appears to be the most effective. A fish which dies in distilled water in a few minutes, lives for weeks in river water, which always contains the necessary saline ingredients. In the present researches the inquiry is extended in order to ascertain whether the same is true for the development of frog's spawn and the growth of tadpoles, and it was found that it is so. Placed in distilled water they die in a few hours; the addition of sodium bicarbonate, or of lime water, or calcium chloride, is insufficient to maintain life. Calcium hydrogen carbonate and tribasic calcium phosphate, on the other hand, sustain life for a considerable time, and development goes on. It seems that those salts of lime, where the base is least saturated by the acid, are those most capable of sustaining function.

HUMAN CHYLE. By D. Noel Paton. (*J. Physiol.*, 11, 109; *Jour. Chem. Soc.*, 1890, p. 394.) In this case the chyle was obtained from a patient in whom the thoracic duct had been cut in an operation for the removal of a tumor of the neck. Systematic observations could only be made at the time when the patient was in an emaciated and dying condition; but from these it was roughly estimated that in health the flow must have been between 3,000 and 4,700 cubic centimetres in twenty-four hours. Four specimens were obtained for analysis, the results of which are given in the following table, in parts per 1,000:

| | | |
|---|---|---|
| Water | 943 | 958 |
| Solids | 56 | 41 |
| Inorganic | 6.2 | 6.7 |
| Organic | 35 | 50 |
| Proteids | 11.8 | 13.7 |
| Fats | 24 | |
| Cholesterin | 0.6 | |
| Lecithin | 0.36 | |

The percentage of solids gradually decreased as the patient became weaker. The proteids are present in smaller quantity than in previously recorded analyses. The percentage of fat is high, but is due to the use of a diet rich in fat.

INFLUENCE OF SODIUM PHOSPHATE ON THE EXCRETION OF URIC ACID. By A. Haig. (*Medico-Chir. Trans.*, 72, 399; *Jour. Chem. Soc.*, 1890, p. 397.) Disodium hydrogen phosphate is a well-known solvent of uric acid, and increases the excretion of that substance. When, however, commercial preparations of the phosphate were employed in cases of gout, it was found that attacks were precipitated, and both in these cases and in healthy persons the amount of uric acid excreted was diminished. Analysis showed these preparations to contain 1.2 to 6.8 per cent. of sodium sulphate, and it is this impurity which causes the retention of the uric acid. The pure phosphate acts as stated at the commencement of this abstract.

The addition of a little sodium hydrogen carbonate increases the efficiency of the phosphate, while the addition of acid (such as dilute phosporic acid) diminishes it. As the acid phosphate is a frequent impurity of the commercial preparation, and as it diminishes the excretion of uric acid, the *pure disodium hydrogen phosphate* should be employed.

ACETONURIA AND DIABETIC COMA. By S. West. (*Medico-Chir. Trans.*, 72, 91; *Jour. Chem. Soc.*, 1890, p. 399.) The author made a systematic examination for acetone of the urine, of both healthy people, and those suffering from various complaints, and of those suffering from diabetes. The results of the investigation are as follows: *Acetone* is absent, or almost so in healthy urine. Acetonuria is common in diabetes without coma, and is not constantly present in cases of diabetic coma. It varies greatly in the same case from time to time, and without apparent cause. It stands in no relation to the amount of sugar in the urine, but varies independently of variations in the sugar and specific gravity. It is often found in other diseases than diabetes. The author having found it in four cases of pneumonia, one case of cirrhosis of the liver, in one of spinal affection, in one of cerebral hemorrhage, and in one of delirium tremens.

The iron reaction[1], on the other hand, is rare, except in case of diabetes. It may be present when acetone is absent, or absent when acetone is present. They may be both absent or both present at the same time, and neither appears to stand in any definite relation to coma. The presence of acetone in the urine, or the occurrence of the iron reaction is, however, by no means *without clinical significance.* They indicate defective metabolism and that the patient is in a worse condition than when the tests gave negative results. Acetone and the substance which gives the iron reaction may be by-products in the formation of the yet unknown poison, and their presence often indicates the presence of this poison and the onset of coma. The occurrence of the iron reaction should be regarded as more serious than that of acetone.

On the Poisons Produced by Bacteria. By L. Brieger and C. Fränkel. (*Berl. klin. Wochenschrift*, 1890, No. 11; *Ber. Deut. Chem. Ges.*, xxiii., p. 251.) According to the authors the injurious action of pathogenic micro-organisms is due to the transformation products generated during bacterial activity. With this in view a number of scientists have undertaken investigations to determine the character of the substance or substances produced by the activity of the micro-germs. Loeffler, Roux and Yersin believe that the poison produced by the diphtheria-bacillus is an enzym; Hankin considers the poisonous product of bacillus anthracis, and Christmas of staphylococcus aureus to be an albuminose.

The authors of this paper have examined the transformation products obtained from pure cultures of the diphtheria bacillus, and find that the poisonous compound is neither a volatile body nor belongs to the ptomaines; but that it is a peculiar body of albuminous characacter, to which compound they have given the name *Toxalbumin.* This substance is easily soluble in water, is precipitated by alcohol, carbonic acid, concentrated mineral acids, potassium ferrocyanide and acetic acid, copper sulphate, silver nitrate, mercuric chloride; not precipitated by lead acetate, sodium, chloride or sulphate, and magnesium sulphate. It has the composition of *Peptone*; contains sulphur. Toxalbumin loses its poisonous properties upon heating its aqueous solutions to 60°; the perfectly dry substance can be heated to 70° for weeks without losing its poisonous properties. It produces the

1. The iron reaction consists in treating the urine with ferric chloride, by which a deep red-brown color is produced. This change of color was believed to be due to the presence of ethyl aceto-acetate in the diabetic urine. It is at present, however, uncertain whether due to this or some allied compound.—J. A. M.

same characteristic paralyzing effects as the diphtheria bacillus, and near the point of infection produces abscesses and necrosis, but *fails* to cause the formation of the *pseudo-membrane*. The formation of this membrane, therefore, is most probably due to the growth of the bacilli themselves.

The injected poison may remain dormant for weeks or months. If less virulent cultures are used, a less virulent poison is obtained.

